# Structure of the *Mycobacterium smegmatis* α-maltose-1-phosphate synthase GlgM

**DOI:** 10.1107/S2053230X20004343

**Published:** 2020-04-03

**Authors:** Karl Syson, Clare E. M. Stevenson, David M. Lawson, Stephen Bornemann

**Affiliations:** a Biological Chemistry Department, John Innes Centre, Norwich Research Park, Norwich NR4 7UH, United Kingdom

**Keywords:** GlgM, α-maltose-1-phosphate synthase, glycosyltransferase, glycogen, α-glucan

## Abstract

*Mycobacterium tuberculosis* produces glycogen (also known as α-glucan) to help evade human immunity. This pathogen uses the GlgE pathway to generate glycogen rather than the more well known glycogen synthase GlgA pathway, which is absent in this bacterium. Thus, the building block for this glucose polymer is α-maltose-1-phosphate rather than an NDP-glucose donor. One of the routes to α-maltose-1-phosphate is now known to involve the GlgA homologue GlgM, which uses ADP-glucose as a donor and α-glucose-1-phosphate as an acceptor. To help compare GlgA (a GT5 family member) with GlgM enzymes (GT4 family members), the X-ray crystal structure of GlgM from *Mycobacterium smegmatis* was solved to 1.9 Å resolution. While the enzymes shared a GT-B fold and several residues responsible for binding the donor substrate, they differed in some secondary-structural details, particularly in the N-terminal domain, which would be expected to be largely responsible for their different acceptor-substrate specificities.

## Abstract 2

GlgM is responsible for the biosynthesis of α-maltose-1-phosphate, the building block for the third known biosynthetic pathway to glycogen/α-glucan, which is a target for antimycobacterials. Here, the first known structure of GlgM is reported.

## 1. Introduction   

**Figure 1:**
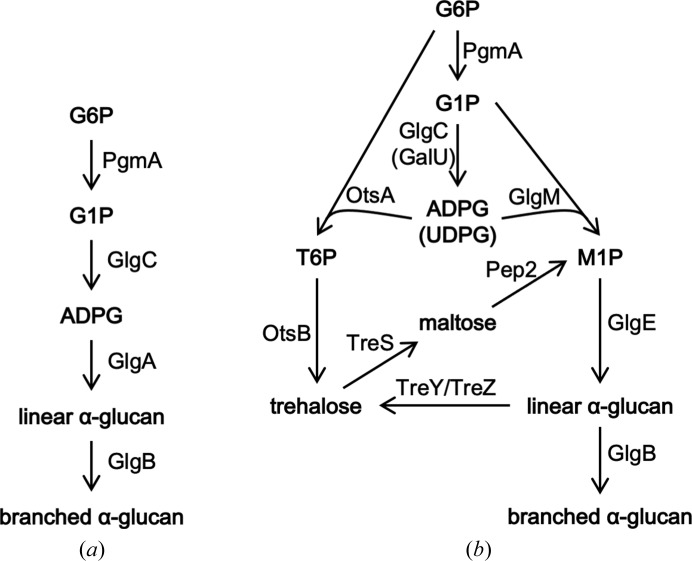
The two bacterial biosynthetic pathways to branched α-glucan (glycogen). (*a*) The well known GlgA-dependent pathway uses ADP-glucose (ADPG) as the building block for the polymerase (for example in *E. coli*). (*b*) The configuration of the pathway in *M. tuberculosis*, which lacks GlgA. The recently discovered GlgE pathway uses α-maltose-1-phosphate (M1P) as the building block for the polymerase. One route to this building block is via GlgM (GT4), a homologue of GlgA (GT5). G6P, glucose-6-phosphate; G1P, glucose-1-phosphate; UDPG, UDP-glucose; T6P, trehalose-6-phosphate. The figure is adapted from Koliwer-Brandl *et al.* (2016 ▸).

Glycogen is a ubiquitous carbon-storage molecule composed of a glucose polymer constructed with α-1,4 linkages and α-1,6 branch points (Preiss, 2009 ▸). The polymerization of glycogen was long thought to be catalysed solely by glycogen synthases that use NDP-glucose as a donor. The bacterial enzyme uses ADP-glucose as the sugar donor [GlgA, GT5 family (Lombard *et al.*, 2014 ▸), EC 2.4.1.21, ADP-α-d-glucose:(1→4)-α-d-glucan 4-α-d-glucosyltransferase; Fig. 1 ▸ *a*], while the eukaryotic enzyme uses UDP-glucose (GT3 family, EC 2.4.1.11). However, another bacterial polymerase has been identified that does not utilize an NDP-glucose donor. GlgE uses α-maltose-1-phosphate as the donor of a disaccharide (Fig. 1 ▸ *b*; Kalscheuer *et al.*, 2010 ▸; Bornemann, 2016 ▸). All three polymerases use a GlgB branching enzyme to generate the mature branched polymer in conjunction with the appropriate polymerase. It is the combination of the polymerase and the branching enzyme that dictates the exact properties of the mature polymer (Rashid *et al.*, 2016 ▸). Importantly, the polymer generated by *Mycobacterium tuberculosis* (often referred to by the generic term α-glucan) has been shown to be important in pathogenesis through its interaction with immune receptors (Koliwer-Brandl *et al.*, 2016 ▸; Kalscheuer *et al.*, 2019 ▸).

An initial estimate of the occurrence of each bacterial pathway, based on genome sequences, suggested that 32% and 14% of bacteria possess the GlgA-dependent and GlgE-dependent pathways, respectively (Chandra *et al.*, 2011 ▸). However, the figure for the former had to be revised down when it became clear that about a third of the GlgA homologues were not GT5 family members but belonged to GT4 (Henrissat *et al.*, 2002 ▸; Koliwer-Brandl *et al.*, 2016 ▸). Indeed, whenever a Gram-positive bacterium possessed the *glgE* gene, the GlgA homologue was invariably a GT4 family member. The significance of this was apparent when the GlgA homologue from *M. tuberculosis* was shown not to possess glycogen synthase activity but α-maltose-1-phosphate synthase activity [EC 2.4.1.342, ADP-α-d-glucose:α-d-glucose-1-phosphate 4-α-d-glucosyltransferase (configuration retaining)]. Thus, the enzyme uses ADP-glucose as the donor and α-glucose-1-phosphate as the acceptor. UDP-glucose can also be used as a donor, but less efficiently. This previously unreported enzyme activity was then named GlgM (Fig. 1 ▸ *b*). It was subsequently necessary to revise the occurrence of the GlgA pathway in bacteria down to 20%.

The configuration of the α-glucan (glycogen) metabolic pathways in *M. tuberculosis* is now known to be very different to that assumed a few years ago (Koliwer-Brandl *et al.*, 2016 ▸). A key feature is that the polymer is generated solely by the GlgE pathway and that its building block is generated either by the phosphorylation of maltose or by the action of GlgM (Fig. 1 ▸ *b*). There were no reported structures of GlgM, so the aim of this study was to elucidate one from mycobacteria.

## 2. Methods   

### 2.1. Protein production   

The *glgM* gene (MSMEG_5080) from *M. smegmatis* mc^2^155 was synthesized with optimum codon usage for expression in *Escherichia coli* (GenScript, Piscataway, New Jersey, USA), allowing expression with a 6×His tag and a TEV cleavage site at the N-terminus of the protein. The construct was ligated into a pET-21a expression vector (Novagen, Darmstadt, Germany) using the NdeI and BamHI restriction sites. GlgM was produced in *E. coli* BL21 Star cells (AMS Biotechnology Europe) which were grown at 18°C to an OD_600_ of 0.6 in lysogeny broth (LB), when expression was induced with 0.5 m*M* isopropyl β-d-1-thiogalactopyranoside (IPTG). The cells were harvested after a further 16 h of incubation. The protein was purified using a 1 ml HisTrap FF column (GE Healthcare, Amersham, United Kingdom) with imidazole-gradient elution. The protein was dialysed against 20 m*M* Tris pH 8.5 and concentrated to 12 mg ml^−1^. Aliquots were stored at −80°C. Size-exclusion chromatography was carried out using a Superdex 200 10/300 GL column eluted with 50 m*M* bis-Tris pH 6.0 containing 5 m*M* MgCl_2_ and 100 m*M* NaCl.

### 2.2. Enzyme assay   

GlgM activity was monitored by following ADP release using a continuous enzyme-coupled spectrophotometric assay involving the oxidation of NADH (Koliwer-Brandl *et al.*, 2016 ▸). Chemicals were purchased from Sigma–Aldrich. Unless otherwise stated, enzyme assays were carried out at 37°C in 50 m*M* bis-Tris propane pH 6.0 containing 5 m*M* MgCl_2_, 0.3 m*M* NADH, 1 m*M* phosphoenolpyruvate, 1 U lactate dehydrogenase, 1 U pyruvate kinase and 0.2 mg ml^−1^ bovine serum albumin. Saturation kinetics for ADP-glucose and α-glucose-1-phosphate were measured in a Costar 96-well plate using a BMG Clariostar plate reader. Eight concentrations each of ADP-glucose and α-glucose-1-phosphate were used from 0.06 to 8.0 m*M*. The effect of pH was measured using sodium/potassium phosphate pH 5.0, bis-Tris pH 6.0 and bis-Tris propane pH 7.0, 8.0 and 9.0, with 1 m*M* each of ADP-glucose and α-glucose-1-phosphate. Temperature dependence (25–50°C) was also determined with 1 m*M* each of ADP-glucose and α-glucose-1-phosphate using a Perkin Elmer Lambda 25 spectrophotometer. Enzyme concentrations were set to allow reactions to progress linearly for 5 min in a cuvette or for 100 s in a plate reader, with the total donor consumption being <10%. Initial rates (*v*_0_/[E]) were determined by monitoring the absorption at 340 nm.

Kinetics curves were fitted to either a Michaelis–Menten (1) or a substrate-inhibition model (2):
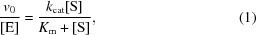


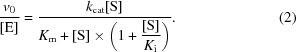



### 2.3. Crystallization and data collection   

Crystallization experiments were performed using a protein concentration of approximately 12 mg ml^−1^ in 20 m*M* Tris pH 8.5 and at a temperature of 20°C. Screening was conducted by sitting-drop vapour diffusion in MRC 96-well crystallization plates (Molecular Dimensions) with a mixture of 0.3 µl precipitant (from both commercial and in-house screens) and 0.3 µl protein solution, using either an OryxNano or an Oryx8 crystallization robot (Douglas Instruments). A variety of commercially available screens were set up and promising conditions were optimized with the latter robot using the same crystallization format. Suitable crystals were grown from 25%(*w*/*v*) PEG 3350, 0.2 *M* malonate, 100 m*M* bis-Tris propane pH 6.5 and were subsequently cryoprotected using the crystallization conditions supplemented with 15%(*v*/*v*) ethylene glycol. Heavy-atom-derivative crystals were prepared by soaking for 90 min in the same cryoprotectant solution containing approximately 1 m*M* HgCl_2_ and were then back-soaked for a few seconds in cryoprotectant lacking the heavy atom.

Crystals were harvested and flash-cooled in liquid nitrogen using LithoLoops (Molecular Dimensions). The mounted crystals were stored in Unipuck cassettes (MiTeGen) prior to transport to Diamond Light Source (DLS), Oxfordshire, United Kingdom, where they were transferred robotically to the goniostat on beamline I03 or I04 and maintained at −173°C with a Cryojet cryocooler (Oxford Instruments). For the best native data set, a single pass of 3600 × 0.1° images was collected with the detector set to a resolution of 1.8 Å at a wavelength of 0.9795 Å. For the derivative data collections, the wavelength was set to 1.0052 Å, which is 50 eV above the theoretical *L*_III_ X-ray absorption edge for mercury. The best derivative data set comprised two consecutive passes of 7200 × 0.1° images collected with the detector set to resolutions of 3.5 and 3.7 Å, respectively. X-ray diffraction data were recorded using a PILATUS 6M hybrid photon-counting detector (Dectris) and were integrated using *XDS* (Kabsch, 2010 ▸) and scaled and merged using *AIMLESS* (Evans & Murshudov, 2013 ▸) via the *xia*2 system (Winter, 2010 ▸); the resultant data-collection statistics are summarized in Table 1 ▸. All of the crystals belonged to space group *P*2_1_2_1_2, but the unit-cell parameters showed considerable crystal-to-crystal variation. For the best native and derivative data sets, these were *a* = 135.37, *b* = 144.93, *c* = 46.47 Å and *a* = 126.35, *b* = 137.45, *c* = 48.89 Å, respectively. Nevertheless, crystal-content estimates suggested that both crystal forms would contain two copies of the 43 485.3 Da protomer (calculated from the native GlgM sequence plus the uncleaved affinity tag) per asymmetric unit, giving solvent contents of approximately 53% and 50% for native and the derivative, respectively.

### 2.4. Structure solution and refinement   

All subsequent steps were implemented via the *CCP*4*i*2 GUI (Potterton *et al.*, 2018 ▸). Initial attempts to solve the structure by molecular replacement with a variety of templates did not yield convincing solutions, most likely owing to the relatively low amino-acid sequence identities of the templates used compared with GlgM (for example 27% for *Pyrococcus abyssi* GlgA; PDB entries 3fro and 2bis; Díaz *et al.*, 2011 ▸; Horcajada *et al.*, 2006 ▸). The structure of GlgM was subsequently solved at 3.5 Å resolution by single-wavelength anomalous dispersion using the Hg-derivative data set through the *CRANK*2 pipeline (Skubák & Pannu, 2013 ▸), which located 12 sites, but the density-modification stage performed by *Parrot* (Cowtan, 2010 ▸) did not detect any twofold non­crystallographic symmetry. In the final stage, *Buccaneer* (Cowtan, 2006 ▸) built a very incomplete and highly fragmented preliminary model. Nevertheless, the two longest fragments appeared to belong to a putative two-domain subunit. These were extracted from the model by editing in *Coot* (Emsley *et al.*, 2010 ▸) and accounted for 61% of the residues expected for the subunit. This coordinate file was converted to a polyalanine model and then split into separate domains. These were used as inputs for a *Phaser* (McCoy *et al.*, 2007 ▸) molecular-replacement job, together with the higher resolution native data set, which was able to place two copies of each domain, giving *R*_work_ and *R*_free_ values of 0.511 and 0.531, respectively, to 1.9 Å resolution. Further refinement with *REFMAC*5 (Murshudov *et al.*, 2011 ▸) did not yield any significant improvement (*R*_work_ and *R*_free_ values of 0.494 and 0.530, respectively). The model was then completely rebuilt with *Buccaneer* to give an 83% complete model with *R*_work_ and *R*_free_ values of 0.284 and 0.313, respectively, to 1.9 Å resolution. Thereafter, the model was completed by several iterations of manual rebuilding in *Coot* and restrained refinement in *REFMAC*5; TLS group definitions obtained from the *TLSMD* server (http://skuld.bmsc.washington.edu/~tlsmd/; Painter & Merritt, 2006 ▸) were used in the later stages of refinement. The geometry of the final model was validated with *MolProbity* (Chen *et al.*, 2010 ▸) and *B*-factor information was extracted via the validation tool in *CCP*4*i*2 before submission to the Protein Data Bank (see Table 1 ▸ for a summary of model statistics).

## 3. Results and discussion   

### 3.1. Enzyme activity of GlgM from *M. smegmatis*   

Attempts to crystallize the α-maltose-1-phosphate synthase GlgM from *M. tuberculosis* were unsuccessful. We therefore switched our focus to the GlgM homologue from *M. smegmatis*, which shares 77% identity with the enzyme from *M. tuberculosis*. The recombinant protein exhibited α-maltose-1-phosphate synthase activity as expected. The pH and temperature optima were 6.0 and 40°C, respectively. The *k*_cat_ values for both α-glucose-1-phosphate and ADP-glucose were an order of magnitude higher than for the *M. tuberculosis* enzyme (Koliwer-Brandl *et al.*, 2016 ▸), while the corresponding values of *K*_m_ were broadly similar (Table 2 ▸). The kinetics of the enzyme conformed to a ternary-complex mechanism. Furthermore, they were consistent with those of the *M. tuberculosis* enzyme, which exhibits a compulsory-order ternary-complex mechanism, whereby ADP-glucose binds to the enzyme before α-glucose-1-phosphate. For example, the kinetics could be fitted to the Michaelis–Menten equation (1) when varying the ADP-glucose concentration with a fixed α-glucose-1-phosphate concentration. In addition, the *k*_cat_ for ADP-glucose increased and then decreased as the fixed α-glucose-1-phosphate concentration increased. In other words, α-glucose-1-phosphate exhibited substrate inhibition at high concentrations, as was manifest when varying its concentration with a fixed ADP-glucose concentration. Inhibition would be expected to occur when α-glucose-1-phosphate unproductively binds before the ADP–enzyme complex dissociates in the last step of the catalytic cycle. Finally, the *K*_m_ and *K*_i_ for α-glucose-1-phosphate were broadly independent of the ADP-glucose concentration and were within an order of magnitude of each other. Although the *M. tuberculosis* GlgM enzyme exhibited a trace of glycogen synthase activity, which was three orders of magnitude lower than its α-maltose-1-phosphate synthase activity (Koliwer-Brandl *et al.*, 2016 ▸), the *M. smegmatis* enzyme exhibited no detectable glycogen synthase activity. In conclusion, GlgM from *M. smegmatis* exhibits high α-maltose-1-phosphate synthase (EC 2.4.1.342) activity.

### 3.2. Structure of the α-maltose-1-phosphate synthase GlgM   

**Figure 2:**
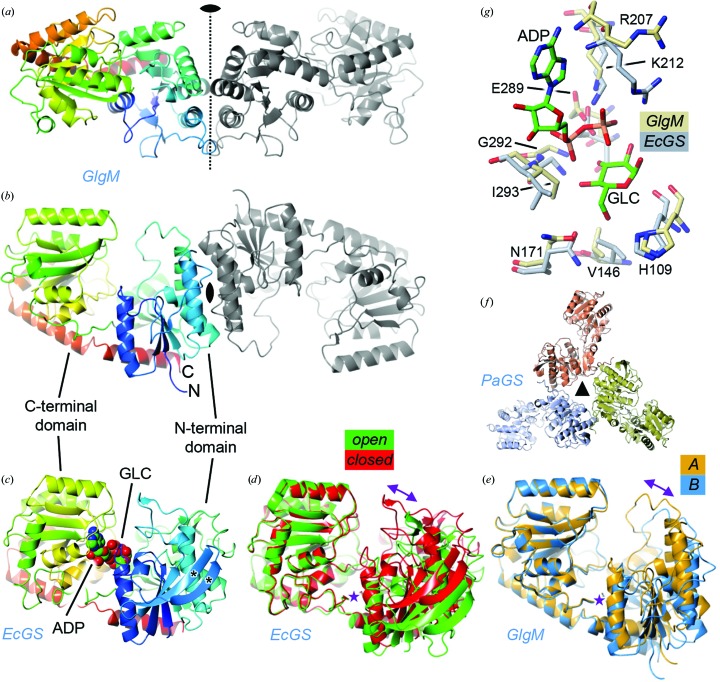
Structure of GlgM from *M. smegmatis* and comparison with bacterial glycogen synthases. (*a*, *b*) Orthogonal views of the GlgM homodimer depicted in cartoon representation with the noncrystallographic twofold axis running vertically and into the screen, respectively (black symbol); the left-hand subunit is in rainbow colours from blue at the N-terminus through to red at the C-terminus and the right-hand subunit is shown in grey. (*c*) Structure of monomeric *E. coli* glycogen synthase (EcGS; PDB entry 2qzs) depicted to allow comparison with the left-hand GlgM subunit in (*b*). Also shown, as van der Waals spheres, are the ADP and α-d-glucose (GLC) ligands bound to EcGS. The asterisks indicate the two β-strands that are not present in the central β-sheet of the N-terminal domain in GlgM. (*d*, *e*) Conformational differences between open (PDB entry 3d1j) and closed (PDB entry 2qzs) states of EcGS compared with the differences between the two protomers of the GlgM homodimer. In both panels, the structures were superposed on the C-­terminal domain and thus emphasize the shift in the N-terminal domain, which is indicated by the two-headed purple arrow; the purple asterisk marks the approximate pivot point. (*f*) Structure of trimeric *P. abyssi* glycogen synthase (PaGS; PDB entry 3fro) as viewed down the noncrystallographic threefold axis (black symbol) and displayed on a smaller scale with respect to the other images. (*g*) Close-up of the conserved GlgM (cream C atoms) and EcGS (grey C atomss; PDB entry 2qzs) donor-binding site displaying a superposition of structurally equivalent key residues (labels refer to GlgM only; see the main text for *E. coli* numbering). Also shown are the ADP and α-d-glucose (GLC) ligands (green C atoms) bound to EcGS. The figures were prepared using *CCP*4*mg* (McNicholas *et al.*, 2011 ▸).

As expected for a GT4 family member, the GlgM protomer comprised two domains, each with an α/β architecture resembling a Rossmann fold (Figs. 2 ▸ *a* and 2 ▸ *b*). All but two of the N-terminal His-tag amino acids were unresolved. Using *DALI* (Holm & Rosenström, 2010 ▸), the nearest known structural homologues to GlgM were identified. These were invariably fellow glycosyltransferases. The lowest r.m.s.d. of the hits was 2.4 Å (PDB entry 3mbo; *Bacillus subtilis* str. Sterne UDP-GlcNAc:l-malate α-*N*-acetylglucosaminyltransferase; BshA; Parsonage *et al.*, 2010 ▸) and the highest protein sequence identity was only 28% (PDB entry 3c4v; *Corynebacterium glutamicum* UDP-GlcNAc:1-l-*myo*-inositol-1-P α-*N*-acetylglucoaminyltransferase; MshA; Vetting *et al.*, 2008 ▸). Both of these hits were GT4 family members, as expected, but neither hit used the same donor or acceptor as GlgM.

The top hits also included GT5 family bacterial glycogen synthases from *E. coli* (PDB entry 2qzs; Fig. 2 ▸ *c*; Sheng *et al.*, 2009 ▸), *Pyrococcus abyssi* (PDB entries 3fro and 2bis; Díaz *et al.*, 2011 ▸; Horcajada *et al.*, 2006 ▸) and *Agrobacterium tumefaciens* (PDB entry 1rzu; Buschiazzo *et al.*, 2004 ▸). The lowest r.m.s.d. was 2.6 Å and the highest protein sequence identity was 27%, both of which were for the *P. abyssi* enzyme, and were very similar values to those of the best GT4 hits. Like GlgM, these enzymes use ADP-glucose as a donor. The structural similarity between these proteins confirmed that GlgM had a GT-B fold. This was to be anticipated because both GT4 and GT5 family members are expected to have this fold according to the Carbohydrate Active Enzymes database (Lombard *et al.*, 2014 ▸).

The glycogen synthases are known to exist in either open or closed conformations, which generally correspond to states with vacant and occupied active sites, respectively. However, these differing states did not result in large domain movements for the *E. coli* (Sheng *et al.*, 2009 ▸; Fig. 2 ▸ *d*) and *P. abyssi* (Díaz *et al.*, 2012 ▸) enzymes. A comparison of the two copies of GlgM within the asymmetric unit (Fig. 2 ▸ *e*) revealed some domain flexibility (r.m.s.d. of 1.774 Å), with inter-domain motion along a similar trajectory to that seen in the *E. coli* enzymes. The two conformations of GlgM were intermediate between the open and closed states observed for the *E. coli* enzyme (for example, PDB entries 3d1j and 2qzs; Sheng *et al.*, 2009 ▸) and within the same *P. abyssi* structure (PDB entry 3fro), although the *A* chain was closest to a closed conformation and the *B* chain was closest to an open conformation. It is likely that there is much more flexibility in this protein family in solution and that the conformations observed in crystal structures are determined in a large part by crystal-packing forces, especially when the active sites are vacant.

Analysis of all inter-subunit contacts present within the GlgM crystal using the *jsPISA* server (http://www.ccp4.ac.uk/pisa/; Krissinel, 2015 ▸) revealed one significant interface of 1041 Å^2^, in which three parallel α-helices from the N-terminal domain of one subunit interact in an antiparallel fashion with the equivalent helices in a noncrystallographic twofold-related subunit to give a six-helix bundle and an elongated homodimer (Figs. 2 ▸ *a* and 2 ▸ *b*). Structural differences in the equivalent regions of the bacterial glycogen synthases would preclude the formation of such an interface. Indeed, the *E. coli* enzyme is reported to be monomeric (PDB entry 2qzs; Fig. 2 ▸ *c*; Sheng *et al.*, 2009 ▸), whilst the *P. abysii* enzyme is trimeric in solution (Horcajada *et al.*, 2006 ▸) and within crystals (PDB entry 3fro; Fig. 2 ▸ *f*; Díaz *et al.*, 2011 ▸). In the trimer, the interactions are also through the N-terminal domains, but in a manner that is distinct from that seen in GlgM (compare Figs. 2 ▸ *a* and 2 ▸ *b* with Fig. 2 ▸ *f*). Confirmation that GlgM forms a dimer in solution was obtained using size-exclusion chromatography. This indicated a molecular mass of 107 kDa, which is close to the theoretical mass of a dimer (87 kDa); the slight overestimation of this value could be owing to the elongated aspect of the GlgM homodimer.

The C-terminal domains of the glycogen synthases are known to be primarily responsible for binding their nucleotide sugar phosphate donors. There were indeed fewer secondary-structural differences between GlgM and the glycogen synthases in this domain (compare the left-hand coloured domain in Figs. 2 ▸ *b* and 2 ▸ *c*), as expected given their shared donor substrates. Several amino-acid side chains that are known to interact with ADP-glucose in the *E. coli* and *A. tumefaciens* glycogen synthases are conserved in GlgM and in the glycogen synthase from *P. abyssi* (Fig. 2 ▸ *g*). These are His109 (His161 in the *E. coli* enzyme), Val146 (Val211), Asn171 (Asn246), Arg207 (Arg300), Lys212 (Lys305), Glu289 (Glu377), Gly292 (Gly380) and Ile293 (Leu381). All of these residues are shared between the GlgM enzymes from *M. smegmatis* and *M. tuberculosis*.

By contrast, the N-terminal domain is likely to be largely responsible for binding the acceptor substrate, and there were more significant differences in secondary structure; most notably, the central parallel β-sheet is two strands shorter in GlgM relative to the bacterial GlgA glycogen synthases (compare the right-hand coloured domain in Figs. 2 ▸ *b* and 2 ▸ *c*). This would be expected given the significant differences between α-glucose-1-phosphate and a polymeric glucan substrate. The acceptor-binding sites of the glycogen synthases have not yet been defined in detail. Likewise, we have been unable to date to obtain ligand-bound structures of GlgM, making it difficult to rationalize these structural differences with respect to function. For example, the soaking of crystals with either ADP-glucose, glucose-1-phosphate, ADP-glucose plus glucose-1-phosphate or ADP plus glucose-1-phosphate did not yield ligand-bound structures. Furthermore, co-crystallization with both ADP and glucose-1-phosphate did not yield suitable crystals.

In conclusion, we have shown that GlgM from *M. smegmatis* exhibits high α-maltose-1-phosphate synthase activity and that it shares a GT-B fold with bacterial glycogen synthases. Further studies will be required to establish which structural features define the specificity for the acceptor in this enzyme.

## Supplementary Material

PDB reference: GlgM, 6tvp

## Figures and Tables

**Table 1:** Summary of X-ray data and model parameters for GlgM Values in parentheses are for the outer resolution shell.

Data set	Hg derivative	Native
Data collection		
Beamline	I03, DLS	I04, DLS
Wavelength (Å)	1.0052	0.9795
Detector	PILATUS 6M	PILATUS 6M
Resolution range (Å)	137.45–3.50 (3.59–3.50)	72.46–1.90 (1.94–1.90)
Space group	*P*2_1_2_1_2	*P*2_1_2_1_2
Unit-cell parameters (Å)	*a* = 126.35, *b* = 137.45, *c* = 48.89	*a* = 135.37, *b* = 144.93, *c* = 46.47
Total No. of measured intensities	579814 (39254)	973029 (59099)
Unique reflections	11345 (810)	73208 (4488)
Multiplicity	51.1 (48.5)	13.3 (13.2)
Mean *I*/σ(*I*)	12.2 (3.9)	18.8 (1.4)
Completeness (%)	100.0 (99.9)	96.3 (95.8)
*R*_merge_ †	0.382 (1.459)	0.088 (2.102)
*R*_meas_ ‡	0.386 (1.474)	0.092 (2.187)
CC_1/2_ §	0.998 (0.870)	1.000 (0.520)
Wilson *B* factor (Å^2^)	84.8	31.5
Refinement		
Resolution range (Å)	—	72.46–1.90
Reflections: working/free¶	—	69344/3792
*R*_work_/*R*_free_ ††	—	0.173/0.212
Ramachandran plot‡‡		
Favoured (%)	—	97.4
Allowed (%)	—	1.5
Disallowed (%)	—	0.1
R.m.s. deviations		
Bond distances (Å)	—	0.010
Bond angles (°)	—	1.581
No. of protein residues	—	*A* chain, 386; *B* chain, 389
No. of waters/sodiums	—	452/2
Mean *B* factors (Å^2^)		
Protein	—	42.5
Ligands	—	46.6
Waters	—	46.2
PDB code		6tvp

† *R*_merge_ = 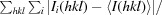 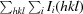.

‡ *R*_meas_ = 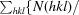 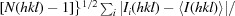 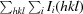, where *I*_*i*_(*hkl*) is the *i*th observation of reflection *hkl*, 〈*I*(*hkl*)〉 is the weighted average intensity for all observations *i* of reflection *hkl* and *N*(*hkl*) is the number of observations of reflection *hkl*.

§ CC_1/2_ is the correlation coefficient between symmetry-equivalent intensities from random halves of the data set.

¶ The data set was split into ‘working’ and ‘free’ sets consisting of 95% and 5% of the data, respectively. The free set was not used for refinement.

†† The *R* factors *R*_work_ and *R*_free_ are calculated as follows: *R* = 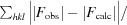 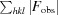, where *F*_obs_ and *F*_calc_ are the observed and calculated structure-factor amplitudes, respectively.

‡‡ As calculated using *MolProbity*.

**Table 2:** Steady-state kinetics of the α-maltose-1-phosphate synthase activity of *M. smegmatis* GlgM na, not applicable.

Fixed [substrate] (m*M*)	*k*_cat_ (s^−1^)	*K*_m_ (m*M*)	*K*_i_ (m*M*)
α-Glucose-1-phosphate			
0.0625	18.6 ± 0.4	0.20 ± 0.02	na
0.125	34.0 ± 0.7	0.19 ± 0.02	na
0.25	69.8 ± 1.2	0.28 ± 0.02	na
0.5	112.6 ± 2.1	0.28 ± 0.02	na
1.0	174.9 ± 3.9	0.41 ± 0.04	na
2.0	178.5 ± 5.0	0.40 ± 0.04	na
4.0	150.3 ± 3.8	0.36 ± 0.04	na
8.0	97.1 ± 2.4	0.22 ± 0.02	na
ADP-glucose			
0.0625	56 ± 8	0.7 ± 0.2	6.2 ± 2.0
0.125	100 ± 14	0.9 ± 0.2	4.0 ± 1.0
0.25	126 ± 9	0.7 ± 0.1	5.5 ± 0.8
0.5	145 ± 11	0.5 ± 0.1	6.0 ± 1.1
1.0	223 ± 24	0.7 ± 0.1	4.6 ± 1.0
2.0	320 ± 33	1.0 ± 0.2	3.1 ± 0.5
4.0	568 ± 85	2.0 ± 0.4	1.5 ± 0.3
8.0	518 ± 79	1.6 ± 0.3	1.9 ± 0.4
